# Green Tea Extracts Epigallocatechin-3-gallate for Different Treatments

**DOI:** 10.1155/2017/5615647

**Published:** 2017-08-13

**Authors:** Chenyu Chu, Jia Deng, Yi Man, Yili Qu

**Affiliations:** ^1^State Key Laboratory of Oral Diseases, West China Hospital of Stomatology, Sichuan University, Chengdu 610041, China; ^2^Department of Oral Implantology, West China Hospital of Stomatology, Sichuan University, Chengdu 610041, China

## Abstract

Epigallocatechin-3-gallate (EGCG), a component extracted from green tea, has been proved to have multiple effects on human pathological and physiological processes, and its mechanisms are discrepant in cancer, vascularity, bone regeneration, and nervous system. Although there are multiple benefits associated with EGCG, more and more challenges are still needed to get through. For example, EGCG shows low bioactivity via oral administration. This review focuses on effects of EGCG, including anti-cancer, antioxidant, anti-inflammatory, anticollagenase, and antifibrosis effects, to express the potential of EGCG and necessity of further studies in this field.

## 1. Introduction

**Figure 1:**
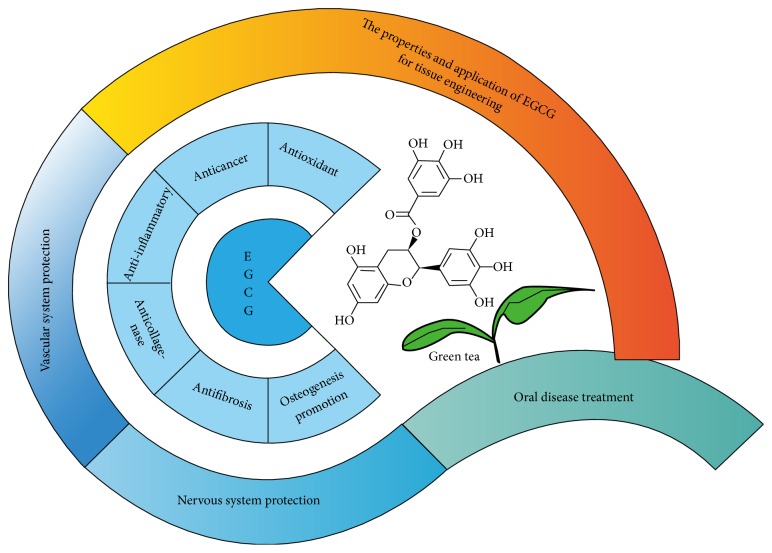
The scheme of properties and application of EGCG.

Green tea is one of the most popular drinks in the area of China and Japan [1], whose main chemical components are tea polyphenols (30% dry weight) [2]. Polyphenols share various therapeutic effects against pathological conditions including cancer, inflammation, diabetes, and cardiovascular diseases [3–5]. Recently, scientific interest in polyphenols has been rapidly increased. Tea polyphenols in green tea possess lots of catechins including catechin, epicatechin (EC), epicatechin-3-gallate (ECG), epigallocatechin (EGC), and epigallocatechin-3-gallate (EGCG). Among these components, EGCG is the most abundant tea polyphenol. Moreover, it is reported that the galloyl moiety of tea catechins plays crucial roles in benefits of tea catechins, especially in lipid lowering effect [6, 7]. Compared to other tea catechins, galloyl moiety of catechins (EGCG and ECG) possesses the most biological activities including angiogenesis [8]. Peoples believe that drinking green tea is beneficial to health and it has been demonstrated that EGCG is with inhibitory effects in many aspects of abnormal changes, such as antioxidant, anticancer, anti-inflammatory, anticollagenase, and antifibrosis effects, appearing in its wide functional range (Figure 1). It can be speculated that EGCG, to some extent, has the effect of protecting organs or tissues from a pile of diseases. Moreover, EGCG has promotional effect on osteogenesis [9, 10]. Although the researches concerning EGCG are still on the road accompanied with quite a few controversies, EGCG is more likely to be beneficial to health.

## 2. The Basic Properties of EGCG

### 2.1. Anticancer Effect

For all the time, the anticancer property of EGCG is the focal point of researches. On one hand, EGCG can inhibit tumorigenesis by inhibiting carcinogen activity [11, 12]. There are findings suggesting that EGCG prevents diethylnitrosamine-induced obesity-related liver tumorigenesis by inhibiting the IGF/IGF-1R axis, improving hyperinsulinemia, and attenuating chronic inflammation [13]. On the other hand, it can restrain tumor proliferation by acting against angiogenesis [14–17]. Shankar et al. found that EGCG inhibits pancreatic cancer orthotopic tumor growth, angiogenesis, and metastasis that are associated with inhibition of PI3K/AKT and ERK pathways and activation of FKHRL1/FOXO3a [15]. Moreover, it can inhibit tumor migration and penetration [18–21] and induce tumor cell death via several mechanisms including caspase-dependent apoptosis, caspase-independent apoptosis, lysosomal membrane permeabilization-mediated cell death, and autophagy. It is widely accepted that hepatocyte growth factor (HGF) is involved in tumor migration and invasion, and EGCG has the capacity to suppress its activity [18–20]. In fact, most of the anticancer effects of EGCG play a role via several signal transduction pathways including JAK/STAT, MAPK, PI3K/AKT, Wnt, and Notch. From the above, it can be easily found that the mechanism of anticancer effect of EGCG is considerably multiple and complicated.

What may be concerned is if this antigrowth effect is also found in normal cells. Park et al. examined the discrepancy of EGCG effects to normal rat osteoblasts (NRO) and human osteosarcoma (MG-63 and Saos-2) [22]. It turned out that after EGCG treatment of micromolar concentrations, the growth and alkaline phosphatase activity of both osteosarcoma cells are inhibited with morphological alterations and G0/G1-phase arrest of the cell cycle, while the NRO is not affected basically. The internal mechanism of the different effects EGCG has on both types of cells remains to be illuminated.

### 2.2. Antioxidant Effect

Antioxidation is a process of vital importance to the health of human body. On the basis of the chemical structure of EGCG, we sort it into antioxidant. The phenol rings in EGCG structure act as electron traps and scavengers of free radicals [23, 24], inhibit the formation of reactive oxygen species, and reduce the harm caused by oxidative stress [25]. It is reported that EGCG can effectively inhibit oxidative stress-induced protein tyrosine nitration induced by oxidative stress in blood platelet [26], and as an antioxidant it can improve the function of mitochondria [27]. However, there are some studies pointing out that EGCG of high concentration can cause self-oxidization and function as the prooxidant [28–31] by producing hydroxyl radicals, hydrogen peroxide, and quinonoid intermediates causing cytotoxicity [32]. For example, Chen et al. found that the catechol-quinone produced by self-oxidation of EGCG and EGC can cross-link with erythrocyte membrane proteins as a cross-linking agent, thus leading to the membrane protein aggregates; herein a galloyl moiety is the essential group of catechins to have the prooxidative effect [33]. Meanwhile in factual physiological concentration (1-2 *μ*M up to 10 *μ*M), EGCG can produce small quantities of reactive oxygen species to activate several signal pathways and then arouse corresponding cellular protective mechanism, thus mainly presenting its antioxidant effects [11, 32]. The complicated biological effects of EGCG may be linked to its products of the metabolism [34].

### 2.3. Anti-Inflammatory Effect

Features of inflammatory response are the symptoms of a large number of immune cell aggregation at the inflammatory sites, release of proinflammatory cytokines, and reactive oxygen/nitrogen (ROS/RNS). ROS/RNS are relative to the activation of transcription factor NF-*κ*B and activator protein- (AP-) 1. After activation, NF-jB and AP-1 will transfer from the cytoplasm to the nucleus and upregulate a variety of inflammatory gene expressions [35], which will give rise to the exacerbated inflammatory response and tissue injury. The anti-inflammatory mechanism is intimate to the process of signal transduction.

EGCG inhibits the transfection of NF-*κ*B and AP-1 to downregulate the expression of iNOS and COX-2 mainly by scavenging NO, peroxynitrite, and other ROS/RNS and decreases the production of inflammatory factors to show the anti-inflammatory effects [36, 37]. IL-8 can stimulate neutrophil aggregation and promote the activation of reactive oxygen species, and it has been pointed out that EGCG could inhibit the IL-8 production of respiratory passage epithelium cell, thereby reducing the severity of respiratory passage inflammatory response [38]. In addition, Tang et al. argued that EGCG downregulates the expression of proinflammatory genes mediated by P2X4 receptor by blocking the JAK1/2 tyrosine kinase signaling pathway in vascular endothelial cells [39]. EGCG plays a crucial role in the regulation of the relative gene expression and transcription, which can account for its anti-inflammatory property.

### 2.4. Anticollagenase Effect

Collagen is a paramount component of the extracellular matrix and of biological characteristics including biodegradability and biocompatibility [40], which have been widely studied in quite many fields. Specific three-helix structure of collagen and stability of collagen structure are closely related to its biological functions. And collagenase can specifically hydrolyze three-dimensional helix structure collagen, destroying its stability, thus affecting its biological function. EGCG-treated collagen can effectively resist the degradation of collagenase [41–43] and maintain three-helix structure in the 37°C. However, mechanisms explaining that EGCG can stabilize the collagen and resist collagenase are still in a controversy. Madhan et al. found that, after EGCG treatment, 95% collagen can resist the degradation of collagenase. They harbor the idea that hydrogen bonding between EGCG and collagenase and hydrophobic interaction inhibit the activity of collagenase, and they observed second structure change of collagenase with round two dichroism spectropolarimeter [42]. But Jackson et al. indicate that EGCG closed collagenase-active site of collagen by being closely conjugated with collagen, which was examined by high performance liquid chromatography and thus resist hydrolysis of collagenase [43]. Therefore, there is a great need of further experiment to explore the intrinsic mechanism explaining that the EGCG can stabilize the collagen structure and resist collagenase degradation.

### 2.5. Antifibrosis Effect

EGCG plays the role of antifibrosis mainly by blocking the transfer of NF-*κ*B from the cytoplasm to the nucleus to downregulate the expression of certain genes. Hepatic fibrosis is a severe complication of chronic liver diseases, which can eventually lead to cirrhosis and even liver failure and liver cancer [44, 45]. Hepatic stellate cells are the major sources of extracellular matrix in chronic liver disease [46], which will be of excessive proliferation and excessive secretion of extracellular matrix during the development of hepatic fibrosis, thus aggravating the liver fibrosis [47, 48]. It is reported that EGCG can inhibit the activation and proliferation of hepatic stellate cells and synthesis of collagen, in a rat model [49–51]. PDGF and IGF play essential roles in the process of liver fibrosis, as EGCG can inhibit the expression of PDGFR and IGF-1R mRNAs, thereby reducing liver fibrosis [52]. In addition, increased expression and activity of MMP-2 are one of the main causes of liver fibrosis, while EGCG can reduce its activity and possess the antifibrosis effect via downregulation of the expression of MMP-2 mRNA [53]. Myocardial fibrosis is mainly due to the increased CTGF in the heart, the promotion of myocardial fibroblast cell proliferation, and excessive secretion of extracellular matrix [54]. Some research shows that, in rat cardiac fibroblasts after AngII stimulation, EGCG could significantly reduce the synthesis of collagen and fibronectin expression and inhibit the cell proliferation. The internal mechanism is that EGCG, by preventing NF-*κ*B transfer to the nucleus and downregulating CTGF gene expression, achieves the effect of antifibrosis [55]. Additionally, the long-term peritoneal dialysis can lead to peritoneal histological aberrance, causing the formation of peritoneal fibrosis. It has been demonstrated that EGCG can also inhibit the NF-*κ*B to reverse the peritoneal fibrosis process [56]. From the information presented above, it can be found that both intracellular reactions and extracellular components can be the aiming targets of EGCG, thus realizing its multiple mechanisms of inhibiting the abnormal changes of the body. Now that the inhibitory effects have been studied, it has promotional influences aiming to make contribution to the health as well.

### 2.6. Osteogenesis Promotion

Osteoporosis is a severe disease characterized by a decrease in bone density and a degeneration in bone fibrous. Epidemiological studies in several countries have found that when more green tea is consumed, the risk of osteoporosis will be lower. Studies have also proved that, EGCG and bone metabolism are closely linked. It can induce the apoptosis of osteoclasts and inhibit the formation of osteoclasts by blocking the generation of NF-*κ*B and IL-1b [57], can also reduce bone absorption by inhibiting osteoclast formation [58], and promote the formation of mineralized bone nodules [59]. The treatment of osteoporosis based on mesenchymal stem cell has captured increasing attention [60]. Bone marrow-derived mesenchymal stem cells possess the ability of self-renewal and differentiation and are considered as ideal cell sources of the treatment of osteoporosis in this way [61, 62]. Jin et al. studied the osteogenic effect of EGCG on hBMSCs by examining cell proliferation, ALP activity, and the expression of related osteogenic markers. The result manifested that EGCG at concentrations of 5 *μ*M significantly promoted the differentiation of hBMSCs into bone cells [9]. It provides the basis for the clinical application of EGCG to rescue osteoporosis by stem cells. Moreover, not only the therapy of stem cells but biomaterials have potential to repair osteoporosis; for example, TCP is one of the ideal scaffolds for bone remodeling. It is bioactive and biodegradable and is capable of space maintaining to improve osteogenesis [63–65]. Animal experiments indicate that a-TCP combining EGCG can significantly stimulate the bone regeneration in skull defects of rats [10], which means the combination of a-TCP and EGCG may be a material having potential to promote bone regeneration. From the above, it can be concluded that EGCG is a promising drug in the treatments or precautions. The properties of EGCG especially the antioxidant and anti-inflammatory ones can present these functions, found during some researches in specific tissues. For specific situations and perhaps the comprehension of several effects mentioned above, we discuss these functions in the next part.

### 2.7. Autoxidation

It is reported that tea polyphenols can be oxidized by superoxide anion (O2^*∙*−^) radicals, which influence the stability. EGCG is not stable and can be oxidized on both the B and the D ring. The main oxidation site of EGCG during autoxidation is the B ring, but the preferred site for O2^*∙*−^ oxidation followed by structural degradation is the D ring. The autoxidation of EGCG further leads to the formation of reactive oxygen species [66–69], which are involved in the biological activities such as telomerase inhibition [69]. A mechanism for the autoxidation of EGCG was based on the formation of EGCG quinone, EGCG dimer quinone, and other related compounds [70]. For example, EGCG can inhibit the angiotensin-converting enzyme (ACE) activity through oxidation into an electrophilic quinone [71], which may be related to the treatment of cardiovascular diseases. Moreover, the formation of autoxidized products also contributes to the inhibition of fibrillation [72]. In addition, EGCG are found to form covalent adducts with cysteinyl thiol residues in proteins through autoxidation to subsequently modulate protein function [73], which can be applied to treat human gastric cancer [74]. The stability and autoxidation of EGCG are also associated with several factors including pH, temperature, metal ion, antioxidant levels, oxygen levels, concentration of EGCG, and other ingredients in tea [66]. It is reported that EGCG is more prone to undergo autoxidation at alkaline pH, and the instability is related to their electrophilic reactivity [75].

## 3. The Application of EGCG in Specific Tissues 

### 3.1. Cancer Treatment

Although the anticancer effect of EGCG in vitro is obvious and aimed at cancerous cells, its effect in vivo and the application in clinical treatment are restricted by several factors. On one hand, the anticancer property of EGCG depends on its high dose, while in normal life the intake quantity of EGCG of our drinking green tea is intensely small and the bioavailability in vivo is low as well. On the other hand, notwithstanding the fact that more and more studies are indicative that EGCG as traditional adjuvant in cancer therapy can reduce the deleterious side effects and get addictive or synergistic effects, EGCG can also cut down the effects of drugs at some extent which limits its clinical application [76]. Therefore, more profound researches are still required in the application of EGCG into clinic.

### 3.2. Oral Disease Treatment

The application of EGCG in the field of oral treatment mainly utilizes its anti-inflammatory effect and inhibiting bone absorption ability. One of the well-known reasons for tooth extraction is pulpal necrosis from bacterial infection, which may induce an inflammatory response and bone resorption at the periapical area of the tooth [77, 78]. Hong et al. studied the healing process of extraction socket with the transplantation of collagenated bovine bone mineral (CBBM) soaked with EGCG, under the condition of injury of the dental periapical of dogs. A research indicated that after the treatment of EGCG, reduced inflammatory response and reduced alveolar bone absorption were observed [79]. Similarly as the dog as the experimental object, Shin et al. found that the experimental group with EGCG is more beneficial to dehiscence defect healing with increased bone remodeling [80]. Additionally, Cho et al. demonstrated that oral administrated EGCG also possesses therapeutic effects on periodontitis triggered by ligation through in vivo experiments [81].

Moreover, EGCG cross-linked collagen membranes have potentials in guided bone regeneration (GBR). GBR is an effective procedure to augment deficient volume of alveolar bone for success of dental implant surgeries, where collagen membranes are usually used [82–85]. To improve the mechanical properties of pure collagen membranes, cross-linking agents are widely used. EGCG can be a potential cross-linking agent for collagen membranes due to its anticollagenase effect [41–43]. Therefore, some studies have also focused on the EGCG-modified collagen membranes. Chu et al. fabricated a novel EGCG-modified collagen membrane with desirable mechanical properties and improved cell adhesion of osteoblasts [86]. However, collagen membranes with the highest concentration of EGCG did not exhibit satisfactory result of cell viability. Previous studies have loaded growth factors on collagen membranes to improve the outcome of GBR [87], and polyethylene glycol (PEG) can be used to improve biocompatibility and decrease cytotoxicity of the added growth factors or drugs. Therefore, PEG was also added onto the EGCG-modified collagen membranes and the results proved that EGCG-modified collagen membranes with PEG modification possessed better biocompatibility and improved cell viability of osteoblasts than those without PEG [88]. To further improve the outcome of bone regeneration, nanosized hydroxylapatite (nano-HA) can also be loaded onto EGCG-modified collagen membranes, which obtained favorable new bone regeneration [89]. However, the results showed that nano-HA-collagen membranes group exhibited less bone regenerative ability than the control group. It is supposed that the imbalance of FBR elicited by implantation of collagen membranes may lead to the loss of bone. Moreover, the new generation of GBR membranes not only should serve as barrier membranes, but also are capable of modulating FBR for bone regeneration after implantation [90]. EGCG-modified collagen membranes can also downregulate the secretion of inflammatory factors [86]. Therefore, EGCG-modified collagen membranes also have great potentials to be immune modulating materials. Further studies on EGCG-modified collagen membranes are still needed.

### 3.3. Nervous System Protection

Neurodegenerative diseases, including Alzheimer's disease (AD), Parkinson's disease (PD), and Huntington's disease (HD), are led by protein misfolding resulting from amyloid protein [91, 92] which is a fibrous polymer rich in *β*  sheets, formed by the self-assembly of proteins of different sequences, structures, and functions [93, 94]. EGCG could conjugate directly with natural peptides not folded to inhibit the formation of toxic intermediate products of *α*-synaptic nucleoprotein and amyloid protein *β* and form a nontoxic and disordered oligomer of these two proteins [95], so as to exert protective effects on nerves. In addition, protein aggregation is generally related to the reduced endogenous antioxidants, inflammation and increased iron ions, NO levels, and other related factors [96–100]. The presence of ROS and RNS will intensify protein misfolding. Experimental studies have emphasized that EGCG could serve as an antioxidant and inhibit the transformation of nitrate and peroxynitrite into NO, thus decreasing ischemic neuronal damage and protecting nerve [36]. Besides the effect on neurodegenerative diseases, EGCG is shown to inhibit the microglia mediated inflammatory response and reduce the damage of the central nervous system triggered by infrasound [101]. In addition, EGCG can promote the healing of extrusion-damaged sciatic nerve, which may be accomplished by changing the expression of the gene controlling apoptosis [102].

### 3.4. Vascular System Protection

Endothelial cells play crucial roles in some physiological processes, including the regulation of vascular tension, coagulation, and permeability. Endothelial cells dysfunction is an essential initial cause of the occurrence of cardiovascular diseases like atherosclerosis. EGCG protects the cardiovascular system and resists all kinds of cardiovascular diseases via its antioxidant and anti-inflammatory function. When atherosclerosis occurs, it accompanies the activation of endothelial cells, the increase of some cytokines, and the expression of adhesion molecule. Ludwig et al. found that EGCG can downregulate vascular cell adhesion protein-1 (VCAM-1) in human umbilical vein endothelial cell, while VCAM-1 plays an important role in atherosclerosis [103]. Perfusion is after myocardial infarct; taking EGCG can reduce neutrophil aggregation and the release of IL-6 and TNF-*α* in order to minimize the endothelial injury induced by ROS [104]. Lorenz et al. found that, after taking green tea, endothelial cells were activated by posttranscriptional regulation (eNOS), resulting in the aortic diastole of rat [105]. The aiming object of EGCG is not limited to endothelial cells. The study found that the proliferation and migration of vascular smooth muscle cell could be inhibited by EGCG, which can induce cell cycle arrest [106]. Cho et al. observed the effect of the scaffold with EGCG on vascular smooth muscle cells and platelets and they found that the scaffold with sustained release of EGCG could inhibit the migration, invasion, and adhesion of VSMCs and activation of platelet. It means that the polymers releasing EGCG can be used to fabricate the EGCG scaffolds and thus prevent vascular restenosis and thrombosis after stent implantation [107]. Although the protective effects of EGCG on the cardiovascular system are proved in numerous experiments, how to apply the EGCG to the corresponding position is still a challenge.

## 4. Future Perspectives

EGCG shows various effects in different cell types in vitro and vivo. Notwithstanding the fact that the properties of the EGCG have been gradually clarified, there still exist quite a few controversies, for example, mechanism of EGCG in collagen stabilization. For the aspect of application, EGCG combined with other drugs for anticancer treatment can possess a synergistic and protective effect. Moreover, EGCG-collagen membranes have great potentials in GBR surgeries. However, EGCG still encounters lots of challenges for clinical application. Oral administration or venous injection of EGCG has low bioavailability, and effects are easily influenced by concentration, derivatives, and other factors. It still needs solutions as to how to deliver EGCG effectively to target sites and protect anticancer drugs from degradation.
